# Paired-like homeodomain 2: a novel therapeutic target for atrial fibrillation?

**DOI:** 10.3389/fgene.2014.00074

**Published:** 2014-04-16

**Authors:** Mengchao Yao, Yujie Cao, Hui Zhu, Yao Chen, Tianhao Zhu, Junjie Xiao

**Affiliations:** ^1^Regeneration Lab and Experimental Center of Life Sciences, School of Life Science, Shanghai UniversityShanghai, China; ^2^Shanghai Key Laboratory of Bio-Energy Crops, School of Life Science, Shanghai UniversityShanghai, China; ^3^Innovative Drug Research Center of Shanghai UniversityShanghai, China

**Keywords:** atrial fibrillation, Pitx2, target, focal activity, reentry

Atrial fibrillation (AF), as a sustained arrhythmia, is featured by uncoordinated atrial activation with the consequent deterioration of mechanical function in the atrium (Mestroni, 2003; Fye, 2006; Fatkin et al., 2007; Otway et al., 2007). A large amount of risk factors for AF have been identified including ageing, male sex, hypertension, ischemic heart disease, myocardial infarction, valvular diseases, and obesity (Benjamin et al., 1994; Krahn et al., 1995; Go et al., 2001). AF can cause several serious complications including congestive heart failure and stroke (Wolf et al., 1991; Benjamin et al., 1998; Kannel et al., 1998; Mestroni, 2003; Piccini et al., 2012). As a most prevalent type of arrhythmia, AF has a life-time risk of 1 in 4 for those people aged over 40 in the United States and Europe (Lloyd-Jones et al., 2004; Donahue et al., 2005; Benjamin et al., 2009; Kim et al., 2011). Almost three million people in the United States are affected by AF, and the number is estimated to be doubled by year 2050 (Roberts and Gollob, 2010). In China, at least 10 million AF patients exist (Zhou and Hu, 2008). Similar trends can also be observed in most other developed and developing countries worldwide (Tsai et al., 2008). AF has become a growing global problem. Dissecting the mechanisms and developing novel effective therapies for AF are highly desirable.

Three classical models have been proposed for the genesis of AF, including focal activity, single-circuit reentry, and multiple-circuit reentry model (Fatkin et al., 2007). The focal-activity hypothesis points out that AF is started and further driven by the rapid firing of single or multiple ectopic foci (Nattel, 2002; Xiao et al., 2011). The multiple-circuit reentry hypothesis assumes that several reentry circuits exist and randomly propagating wave fronts persist in receptive tissue while the single-circuit reentry hypothesis focuses on the interaction of a rotor with irregular waves in the atrium and “fibrillatory conduction,” the spatially variable refractory properties of atrial tissue (Nattel, 2002; Xiao et al., 2011).

Although AF is a most prevalent type of arrhythmia, its genetic etiology remains unclear (Franco et al., 2011). At the very early beginning, AF is generally appreciated to be uninheritable (Xiao et al., 2011). Subsequently, 5% of AF patients and up to 15% of individuals with lone AF have been identified to have a familial history (Christophersen et al., 2013), which encourage the starting of genetic studies in familial form of AF. Besides that, the common variants in AF in the general population have been paid special attention with the recently published results from several large genome-wide association studies (GWAS) (Darbar et al., 2003; Fox et al., 2004; Christophersen et al., 2009; Sinner et al., 2011; Mahida, 2014). A susceptibility locus for AF on chromosome 4q25, which was reported in 2007 in the first GWAS for AF (Gudbjartsson et al., 2007) and has been independently replicated in multiple other association studies (Kaab et al., 2009; Sinner et al., 2011), is of highly interest. The identified signal at the 4q25 locus lies within an intergenic region without any currently known genes (Sinner et al., 2011; Mahida, 2014) and the closest gene, paired-like homeodomain 2 (Pitx2), is considered as a most promising candidate. Pitx2 is located 150,000 bases away and has been reported to contribute to cardiac development (Franco et al., 2011; Liu et al., 2012).

The Pitx2 gene mainly encodes three distinct isoforms including *Pitx2a, Pitx2b*, and *Pitx2c* (Schweickert et al., 2000; Cox et al., 2002). A fourth isoform, *Pitx2d* is also expressed in human, functioning as a dominant negative protein (Cox et al., 2002). Pitx2 has been reported to participate to the establishment of left–right asymmetry of the heart (Lin et al., 1999). In addition, Pitx2 plays a critical role in the development of the left atrium (Campione et al., 2001, 2002; Mommersteeg et al., 2007) and pulmonary vein myocardium as well (Mommersteeg et al., 2007). Interestingly, pulmonary vein is a source of AF (Nattel, 2002). Thus, inactivated Pitx2 may affect the development of pulmonary vein myocardium, contributing to ectopic electric activity from pulmonary vein. The loss-of-function of Pitx2 causes several severe cardiovascular defects, including atrial isomerism, double inlet left ventricle, abnormal aortic arch remodeling and transposition of the great arteries (Franco and Campione, 2003; Wang et al., 2013). Since the published of the identification of Pitx2 as a candidate in the first GWAS study, special attention has been paid to its potential role in AF. Knockout of Pitx2 in mice leads to pro-arrhythmogenic alterations in the action potential (Wang et al., 2010; Chinchilla et al., 2011; Kirchhof et al., 2011). The expression level of Pitx2c, a major form of Pitx2 in the adult heart, has been reported to be impaired in patients with AF (Chinchilla et al., 2011). Decreased Pitx2 leads to atrial electrical and structural remodeling (Chinchilla et al., 2011), both are arrhythmogenic alterations, and can finally increase susceptibility to atrial arrhythmias (Wang et al., 2010; Chinchilla et al., 2011; Kirchhof et al., 2011). Evidences show that the presence of common single nucleotide polymorphism (rs2200733, rs1033464) at the 4q25 locus (near Pitx2) is an independent predictor of AF recurrence after direct current cardioversion (Parvez et al., 2013). Moreover, rs1033464 has also been reported as an independent predictor of successful rhythm control in antiarrhythmic drugs (Parvez et al., 2012). Although the putative function of Pitx2 in the fetal and adult heart is largely unknown, accumulating evidences show that defects in Pitx2 result in defects of cardiac conduction system (Franco et al., 2011), which might be a cause of spreading irregular waves and leads to AF. Promisingly, Pitx2 has been reported to prevent susceptibility to atrial arrhythmias through inhibition of left-sided pacemaker, such as the SAN-specific genetic program in left atrium (Wang et al., 2010), providing a novel therapeutic target for AF. A Pitx2 conditional knockout mouse which deletes Pitx2 in postnatal atrium but leaving the developmental function of Pitx2 intact exhibit the phenotype of irregular R-R interval with disappeared P-wave amplitude, indicating impaired atrial conduction (Tao et al., 2014). Thus, inactivated Pitx2 may also disrupt normal atrial conduction and thereafter promote re-rentrant circuits. Accumulating evidence has indicated that Pitx2 is a promising therapeutic target for AF. Further understanding of the role of Pitx2 in the genesis of AF holds the potential to be eventually developed as a novel effective therapy for this common arrhythmia.

## Conflict of interest statement

The authors declare that the research was conducted in the absence of any commercial or financial relationships that could be construed as a potential conflict of interest.
